# The genetic diversity and population structure of two endemic Amazonian quillwort (*Isoetes* L.) species

**DOI:** 10.7717/peerj.10274

**Published:** 2020-11-09

**Authors:** Mirella Pupo Santos, João V.S. Rabelo Araujo, Arthur V. Sant’anna Lopes, Julio Cesar Fiorio Vettorazzi, Marcela Santana Bastos Boechat, Fernanda AbreuSantana Arêdes, Naiara Viana Campos, Emiliano Nicolas Calderon, Fernando M. Gomes Santos, Tais Nogueira Fernandes, Rodrigo Nunes da Fonseca, Messias Gonzaga Pereira, Guilherme Oliveira, Daniel Basilio Zandonadi, RodrigoLemes Martins, Francisco de Assis Esteves

**Affiliations:** 1 Instituto de Sustentabilidade e Biodiversidade, Universidade Federal do Rio de Janeiro, Macaé, Rio de Janeiro, Brazil; 2 Laboratório de Melhoramento Genético Vegetal, Universidade Estadual do Norte Fluminense, Campos dos Goytacazes, Rio de Janeiro, Brazil; 3 Environmental Studies Office, Vale, Belo Horizonte, Minas Gerais, Brazil; 4 Instituto Tecnológico Vale, Belém, Pará, Brazil

**Keywords:** *Isoetes cangae*, *Isoetes serracarajensis*, ISSR, Lycophyte, Gene flow, Mating system

## Abstract

**Background:**

Two endemic lycophyte species *Isoetes cangae* and *Isoetes serracarajensis* have been recently described in the State of Pará in the Amazon forest located in northern Brazil. *Isoetes* L. has survived through three mass extinctions. Plants are considered small-sized, heterosporous, and can display a great diversity of physiological adaptations to different environments. Thus, the current study aimed to estimate the genetic variation of the populations of *I. cangae* and *I. serracarajensis* to generate information about their different mechanisms for survival at the same geographical location that could point to different reproductive, adaptative and dispersal strategies and should be considered for effective conservation strategies.

**Methods:**

The genetic diversity and population structure of *I. cangae* and *I. serracarajensis* were investigated using Inter Simple Sequence Repeat (ISSR) molecular markers. Total genomic DNA was isolated, and the genetic diversity parameters were calculated.

**Results:**

The sixteen primers produced 115 reproducible bands, 87% of which were polymorphic. A high level of polymorphic loci (81.74% and 68.48%) and a high Shannon index (Sh = 0.376 and 0.289) were observed for *I. cangae* and* I. serracarajensis*, respectively. The coefficient of genetic differentiation between population areas (G_ST_) showed a higher value in *I. serracarajensis* (0.5440). Gene flow was higher in *I. cangae* (1.715) and lower in *I. serracarajensis* populations (0.419). Overall, the results further show that *I. serracarajensis* and *I. cangae* are two species with considerable genetic variation and that these differences may reflect their habitats and modes of reproduction. These results should be considered in the development of effective conservation strategies for both species.

## Introduction

The genus *Isoetes* L. is characterized by approximately 200 species of vascular plants that have been catalogued with molecular dating studies, tracing its evolutionary roots to the Devonian in the Palaeozoic (Pigg, 2001). This genus has survived through three mass extinctions, most likely due to adaptations to large environmental changes over hundreds of millions of years. *Isoetes* are considered small-sized heterosporous plants (Troia et al., 2016), which can display a great diversity of physiological adaptations to different environments (Smolders, Lucassen & Roelofs, 2002). These lycophytes can be associated with aquatic habitats such as seasonally flooded plains and oligotrophic lakes and grasslands (Smolders, Lucassen & Roelofs, 2002; Troia et al., 2016).

The *Isoetes* genus also shows a variety of ploidy levels, which has been suggested to be an important and determining feature for adaptation to several regions of the planet (Liu, Gituru & Wang, 2004; Bucharová & Münzbergová, 2012). Thus, the genus can be considered almost cosmopolitan (Liu, Gituru & Wang, 2004; Troia et al., 2016). Sexual reproduction by self-fertilization and cross-fertilization has also been reported in the genus (Chen et al., 2009; Hoot, Taylor & Napier, 2006; Pant & Srivastava, 1965). Sexual reproduction by cross-fertilization results in high gene flow that is determined by the geographic distance from the genitors and the presence of spore dispersing agents (Takamiya, Watanabe & Ono, 1996; Smolders, Lucassen & Roelofs, 2002; Li et al., 2012).

The species *Isoetes cangae* and *Isoetes serracarajensis,* the targets of the current study, were recently described from the Amazonian ferruginous rocky outcrops of Serra dos Carajás in the southeast of the State of Pará, Northern Brazil (Pereira et al., 2016; Nunes et al., 2018). *I. cangae* is an aquatic plant restricted to Amendoim Lake in the ferruginous plains of the southern region of the Serra dos Carajás, listed as “Critically Endangered” in the IUCN red list (Lansdown, 2019). This species is bisexual with sexual reproduction (Pereira et al., 2016; Caldeira et al., 2019), and the possibility of asexual reproduction should not be excluded. *I. cangae* contrasts with its congener *I. serracarajensis*, which is found in several ferruginous plateaus of southeastern Pará State and is associated with seasonally flooded environments or terrestrial habitats in wet soils. *I. serracarajensis* is bisexual and should be classified as vulnerable (Pereira et al., 2016).

The specific genetic diversity generated is associated with the inherent properties of population colonization and isolation. The mating system directly influences the genetic variation of each species; thus, it is a fundamental factor for population establishment and survival. Genetic diversity can be assessed using molecular markers such as the inter simple sequence repeat (ISSR), a dominant marker with a multiallelic nature, high reproducibility, and large genome coverage. ISSR reveals a large number of polymorphic fragments and thus constitutes a suitable marker of intrapopulation and interpopulation genetic diversity for *Isoetes* (Chen et al., 2005; Gentili et al., 2010; Ma et al., 2019).

In this work, we used 16 ISSR markers to estimate the genetic variation of the only known *I. cangae* population and four *I. serracarajensis* populations in the Brazilian Amazon region. We aimed to estimate (1) the genetic variation of the populations of *I. cangae* and *I. serracarajensis* and (2) the genetic structure and its correlation with geographical distribution. Our findings should be considered for effective conservation strategies.

## Materials & Methods

### Plant materials

**Figure 1:**
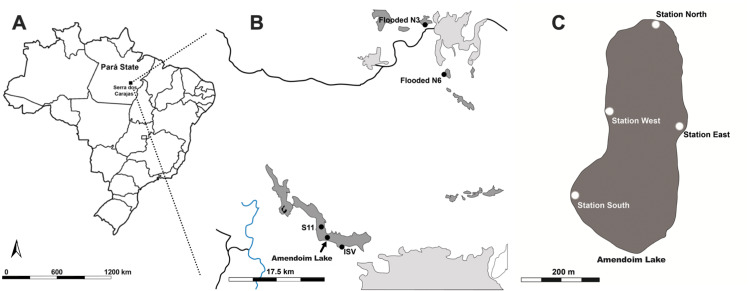
Ferruginous rocky outcrops of the Serra dos Carajás, Pará State, Brazil, Amendoim Lake. (A) Brazil map (B) *I. serracarajensis* was gathered at different regions in the Serra dos Carajás: two locations on the ferruginous plateaus north of the Serra dos Carajás, the flooded areas N3 (ISN3) and N6 (ISN6), and two locations on the ferruginous plateaus of the southern portion, ISV and S11 (IS11). (C) Four collection areas of *I. cangae*, Station North (ICN); Station South (ICS); Station East (ICE); Station West (ICW). Blue lines are rivers, black lines are roads, dark grey indicates Canga areas and light grey indicates human activity.

Specimens of *Isoetes cangae* and *Isoetes serracarajensis* (Pereira et al., 2016) were collected in February 2018 in ferruginous rocky outcrops from Serra dos Carajás, Pará State, Brazil. *I. cangae* inhabits Amendoim Lake’s marginal areas, where the water depth reaches 3 metres (Da Silva et al., 2018). Plants were collected in four areas from a 12.98 ha lake arranged on a ferruginous plateau south of the Serra dos Carajás, known as Serra Sul. The testing areas were named Station northern (ICN), Station southern (ICS), Station eastern (ICE), and Station western (ICW) (Fig. 1B). To evaluate possible genetic differences between points within the lake, individuals were collected from each area. Lake Amendoim is the original location from which the species was described, and is, thus far, the species’ only known location. Fifty individuals were collected in testing areas of the lake, and individuals were collected from each area with a minimum distance of 2.0 metres between them. For each population, we collected at least twelve specimens.

*I. serracarajensis* was collected from different regions in the Serra dos Carajás: two locations on the ferruginous plateaus north of the Serra dos Carajás, N3 and N6; and two locations on the ferruginous plateaus of the southern portion, the ISV marsh and S11 (Fig. 1A). The four sampled populations were defined as ISN3, ISN6, ISV, and IS11, and a total of 40 specimens were collected. All are temporarily flooded areas. For each population, we collected at least ten specimens from each area where the species is found with a minimum distance between each specimen of 2.0 metre. The location information, habitat, area occupied by the population (m^2^), categorized population density, and number of samples are presented in Table 1. Collecting permits were granted by Instituto Chico Mendes de Biodiversidade do Ministério do Meio Ambiente (ICMBio, http://www.icmbio.gov.br) number 59724-2.

**Table 1:** Description of ISSR polymorphic primers validated for the genus ***Isoetes***.

**ID**	**Sequences**	**At (°C)**	**TNL**	**NPL**	**%PPL**
**LB1**	(GA)_6_C	50	8	5	62.5
**LB2**	(GT)_6_C	48	5	4	80
**LB5**	(AG)_8_YT	52	8	7	87.5
**LB6**	(AC)_8_CC	52	9	9	100
**LB11**	(GGAT)_3_GA	50	8	7	87.5
**LB14**	(GA)_8_C	48	7	6	85.7
**LB18**	(AGA) _8_YC	50	10	9	90
**LB19**	(AG)_8_YA	50	7	6	85.7
**LB23**	(AC)_8_YG	48	5	4	80
**LB40**	(AC)_8_(CT)T	48	8	8	100
**LB41**	(TC)_8_(AG)G	52	7	7	100
**LB42**	(TC)_8_(AG)A	48	5	4	80
**LB43**	(GT)_8_(CT)C	52	8	7	87.5
**LB48**	(GA)_8_T	48	6	5	87.5
**LB52**	(ATC)_6_C	48	8	8	100
**LB57**	(GA)_8_AC	48	5	4	80
**Total**			115	100	

**Notes.**

ID Identification of the primers Sequences (5′–3′) At (°C): annealing temperature NPL Number of polymorphic loci TNL Total number of polymorphic loci %PPL Percentage of polymorphic loci per primer

### Estimates of density and distance between collection sites

The density of *I. cangae* was estimated to understand the processes related to the maintenance of the diversity of this species and its restricted occurrence. For density estimation, cover and number of individuals were determined by free diving at approximately 12 points randomly distributed in each of the four sampling areas. At each point, a 1 ×1 m square of plastic (PVC), subdivided into four quadrants of 0.25 m^2^, was arranged randomly on the substrate. In each square, the percentage coverage (percent area of the square occupied) and density estimation (number of individuals per square metre) were estimated. Among fifty squares sampled, counting was performed in fifteen squares. The density category was established as a plus symbol “+” corresponding to an average of 3 plants per square metre. The coordinates (latitude and longitude) for each sample point were recorded with GPS. The areas occupied by the sampled specimens (AOPEA) and respective distances between sampled areas were measured in Google Earth Pro (Google LLC 2019). For the determination of the area, a polygon comprising all the samples of each area was drawn and is represented in m^2^. The smallest distance between the polygons of the different sampling areas was used to establish the distance among them. The maps were made in Adobe Illustrator CS6.

### Total DNA extraction and ISSR PCR amplification

Total genomic DNA was isolated from 1 gram of dry leaves. Leaves were macerated using liquid nitrogen and the extraction was performed with the help of the DNeasy Plant Maxi Kit (Qiagen). The DNA was quantified by Nanodrop, and 50 ng of genomic DNA was used for each PCR. Annealing temperatures varied between 48 °C and 52 °C according to the primer used. Among the twenty tested primers, sixteen were selected for further analysis (Table 2). Integrity assessment and quantification of DNA were performed by 2% agarose gel electrophoresis and Nanodrop (Nanodrop 2000 software), respectively.

### Data analyses

A binary matrix was constructed from the gel analysis. The presence of bands is indicated by the number 1 and the absence of bands by the number 0. Genetic dissimilarity through the weighted index was determined using this binary matrix.

The total number of loci (TNL), number of polymorphic loci (NPL), and percentage of polymorphic loci per primer (PPL%) were calculated with the software GenALEx 6.5 (Peakall & Smouse, 2012). The occurrence of rare loci was estimated as the existence of loci present in only 10% of the studied area. Diversity indexes such as the Shannon index (I), which was calculated as Sh = − ∑pilog2pi, expected heterozygosity (HE), number of alleles (Na), effective number of alleles (Ne), and molecular variance analysis (AMOVA), were calculated with GenALEx 6.5 software. The relative magnitude of differentiation among areas (differentiation indices) [G_ST_ = (HT-HS)/HT] and an estimate of gene flow (Nm) from G_ST_ as Nm = 0.5 (1 - G_ST_)/G_ST_ were determined using POP gene version 1.32 (Wright, 1951; Slatkin, 1985; Slatkin & Barton, 1989; Yeh & Boyle, 1997).

The genetic relationship among populations was demonstrated by creating a dendrogram based on Nei’s (1972) genetic distance using an unweighted pair-group method of cluster analysis that used arithmetic averages **(**UPGMA) in the programme GenALEx 6.5 and MEGA7 (Molecular Evolutionary Genetics Analysis) software (Kumar, Stecher & Tamura, 2016). The correlation between the genetic distance (Nei’s genetic distance) and the geographic distance between the populations was investigated by the Mantel test using XLSTAT software (Addinsoft, Paris, France). The *p*-value was computed using 999 Monte Carlo simulations (Peakall & Smouse, 2012).

## Results

### Genetic diversity

Sixteen primers were used for both species and produced approximately 115 reproducible loci for the genotype (Table 1); at least one hundred one (101) were polymorphic loci (mean of seven loci per primer), which corresponded to almost 87% of the entire sample. The most informative primer set was LB6 with nine polymorphic loci (Table 1). The amplified fragments ranged from 100 to 2,000 bp.

*I. cangae* showed 81.74% polymorphic loci and expected heterozygosity (He = 0.245) (Table 3). For *I. cangae*, the ICW area (Fig. 1; Table 2) displayed the highest percentage of polymorphic loci (64.35%) by primers (PLP) (Table 3), while the lowest percentage was obtained for the ICS area, 33.04%. The expected heterozygosity (He) values ranged from 0.187 in ICW to 0.096 in ICS. The ICW and ICN areas presented a higher number of rare loci (Table 3). The populations of *I. serracarajensis* showed 63.48% polymorphic loci and a diversity index He of 0.187. The population with the highest PLP was ISV (52%) and that, with the lowest was IS11 (18%) (Table 3). Among the populations, ISV displayed the highest expected heterozygosity value (0.164), while ISV displayed the lowest (0.043). The ISN3 and IS11 populations also showed five rare loci, while ISN6 and IS11 had three rare loci (Table 3).

**Table 2:** Identification of the population areas in the state of Pará, northern region of Brazil.

**Species and collection area**	**LS**	**PC**	**Lat/Lon**	**AOP**	**PD**	**SN**
*I. cangae* Amendoim lake Station North	Serra Sul	ICN	6°23′47,95″/50°22′17,05″	1.536	+	12
*I. cangae* Amendoim lake Station South	Serra Sul	ICS	6°24′03,73″/50°22′23,27″	1.041	+++++	11
*I. cangae* Amendoim lake Station East	Serra Sul	ICE	6°23′57,06″/50°22′14,37″	1.188	++++	11
*I. cangae* Amendoim lake Station West	Serra Sul	ICW	6°23′55,84″/50°22′21,01″	782	+	14
*I. serracarajensis* Flooded N3	Serra Norte	ISN3	6°02′44,90″/50°12′34,68″	4.340	++	8
*I. serracarajensis* Flooded N6	Serra Norte	ISN6	6°07′33,97″/50°10′39,43″	99	++	12
*I. serracarajensis* ISV	Serra Sul	ISV	6°24′31,39″/50°21′05,38″	4.959	+++	13
*I. serracarajensis* S11	Serra Sul	IS11	6°22′33,90″/50°23′00,34″	1.561	+	5

**Notes.**

LS Location of Species PC Population Code Lat/Lon Latitude/Longitude AOP Area Occupied by Population (m^2^) PD Population Density

Categories were stablished as follows: one plus signal (+) represents average of 3 plants per m^2^.

SN, Sample Number.

**Table 3:** Genetic diversity among the populations area of *Isoetes cangae***and***I. serracarajensis*.

	**TPL**	**% PLP**	**Rare Locus**	**He**	**Sh**	**G**_**ST**_	**Nm**
***Isoetes cangae*(collection areas)**							
**ICN**	47	42.61	6	0.136	0.208		
**ICS**	36	33.04	3	0.096	0.149		
**ICE**	47	40.87	1	0.123	0.190		
**ICW**	79	64.35	6	0.210	0.318		
**Mean**		41.74	4.5	0.129	0.199		
**Species level**		81.74		0.245	0.376		1.714
***Isoetes serracarajensis*(populations)**							
**ISN3**	25	21.74	5	0.082	0.120		
**ISN6**	32	26.96	3	0.071	0.111		
**ISV**	52	45.22	3	0.164	0.242		
**IS11**	18	16.52	5	0.043	0.060		
**Mean**		24.35	4	0.076	0.115		
**Species level**		63.48		0.187	0.289	0.5440	0.419

**Notes.**

TPL Total polymorphic locus % PLP Percentage of polymorphic loci Rare Locus; He corrected expected Nei heterozygosity (1978) (assuming Hardy–Weinberg equilibrium) Sh Shannon & Weaver Diversity Index GST Proportion of genetic diversity among populations Nm Gene flow

### Genetic structure

**Figure 2:**
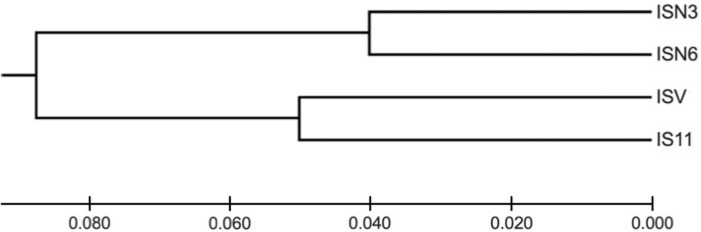
Dendrogram obtained from the dissimilarity matrix based on the Nei genetic distances using an unweighted pair-group method of cluster analysis that used arithmetic averages (UPGMA). ISSR fragments detected in four populations of *I. serracarajensis* (ISN3, ISN6, ISV, IS11).

**Figure 3:**
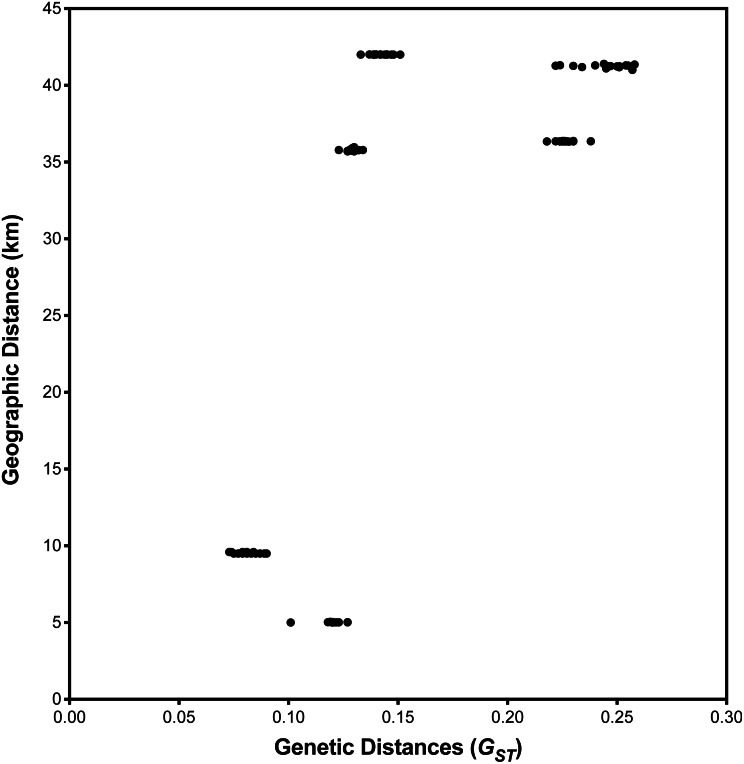
Relationship between geographic and pairwise genetic distances (G_*ST*_) between population. Correlation among the four local population areas of *I. serracarajensis*. There was no correlation between geographic distance (km) and genetic distance among populations with the Mantel test for population of *I. serracarajensis* (*r* =  − 0.5218 *P* = 0.2972).

*I. cangae* presented a high Shannon diversity index value at the species level, Sh = 0.376, and high genetic flow (1.714). The ICW and ICN areas displayed the highest values of Sh = 0.318 and Sh = 0.208, respectively (Table 3). It is interesting to observe that both areas, even with the highest diversity values of Sh, are those with the lowest densities (Table 2). The populations of *I. serracarajensis* displayed Shannon diversity index values (Sh = 0.289), a G_ST_ equal to 0.5440 and low genetic flow (0.419) (Table 3). The ISV population displayed the highest Sh = 0.242, while the lowest value was obtained with the IS11 subpopulation (Sh = 0.06) (Table 3). From the dissimilarity matrix, based on the Nei genetic distances, a dendrogram was generated to investigate area clustering (Fig. 2). Comparison of the total genetic variance by ISSR markers showed that the genetic variance of *I. serracarajensis* was 55% within each population and 45% among the populations (Table 4). A dendrogram was generated based on the Nei genetic distances, and the results show separation in two distinct clusters: the ISN3 and ISN6 populations (Serra Sul) and the ISV and IS11 populations, which occur in the same region (Serra Norte). The correlation between the genetic distance and the geographic distance among the populations showed a significant relationship (Mantel test, *r* = 0.821, *P* < 0.0001) (Fig. 3).

## Discussion

The genetic diversity of a species is a result of evolutionary processes, including recent changes such as habitat reduction, which may lead to a decline in genetic diversity and decrease the survival potential for a given species.

The results of the ISSR marker analysis revealed high numbers of polymorphic loci (81.74%) for *I. cangae*. In genetic structure studies, *I. cangae* presented a high level of intrapopulation genetic diversity (He = 0.245; Sh = 0.376). *I. cangae* is one of the most diverse species in the world. Similarly, high diversity was found in *I. malinverniana* (PLP = 95.33%; He = 0.267; Sh = 0.411) (Gentili et al. 2011), *I. yunguiensis* (PLP = 82.05%; He = 0.291; Sh = 0.434) (Dong et al. 2018) and *I. hydrophila* (PLP = 82%; He = 0.351; Sh = 0.217) (Chen et al., 2005). Similar studies were carried out with other plants, such as the endangered shrub *Elaeagnus macrophylla* (PLP = 48,928%; He = 0.1149; Sh = 0,1848) (Wang et al., 2020) and the endangered aquatic *Ottelia acuminata* var. *jingxiensis* (PLP = 73%; He = 0.441. Sh = 0,781) (Li et al., 2019). The approach used here is still important for a broad conservation effort among diverse plant species.

The prevalence of sexual reproduction explains the genetic diversity found in *I. cangae*. The presence of long-lived individuals and overlapping generations observed may also be a contributing factor to the genetic diversity of this species. Similar results were found in species such as *I. asiatica*, *I. taiwanensis*, and several endangered *Isoetes* and fern species (Small & Hickey, 1997; Kim et al., 2009; Bucharová & Münzbergová, 2012). Studies in seven *Isoetes* species from Brazil suggested a correlation between ploidy level and spore size (Pereira, Mittelbach & Labiak, 2015); therefore, it is tempting to speculate that *I. cangae* is diploid.

The dispersion of *Isoetes* ciliary microspores, which occurs up to a few centimetres from the mother plant, the results in higher genetic similarity between neighbouring individuals and greater diversity in isolated plants (Smolders, Lucassen & Roelofs, 2002; Hoot, Taylor & Napier, 2006; Pant & Srivastava, 1965; Kang, Ye & Huang, 2005). Due to the limited size of Amendoim Lake, spore dispersion can be achieved for the entire area by extrinsic factors with high vagility, such as fish and other animals (alligators and turtles). In addition to spores, sporangia, or even entire sporophylls, can be dispersed over long distances by external factors such as water flow and animals (Small & Hickey, 1997; Chen et al., 2005; Bucharová & Münzbergová, 2012). Thus, it is possible to have continuous spore flow in the lake, enabling free crossing between individuals. This factor can be proven by the high gene flow in *I. cangae* observed in the lake (Table 3). Field evaluations showed fertile plants year-round; therefore, spores can be dispersed in water continuously. In addition, synaptospory in *I. cangae* could be an essential mechanism in dispersal events for this species in a lake, as exemplified by Troia (2016). Self-compatibility and maturity desynchronization of spores in the gametophyte were observed in *I. cangae* by Caldeira et al. (2019) and by our group through in vitro fertilization and seedling production (Fig. S1).

**Table 4:** Analysis of molecular variance (AMOVA) of populations of *I. serracarajensis***via ISSR markers**.

***I. serracarajensis***					
**SV**	**d.f**	**MSD**	**VC**	**%**	*P*-value^*^
Among pops	3	77.612	7.477	45	<0.001
Within pops	34	9.270	9.270	55	<0.001
Total	37		16.747	100	

**Notes.**

SV Sources of variance d.f degrees of freedom MSD mean square displacement VC variance component % Percentage of total

* *P*- value* statistic significance is based on 1,000 permutations.

The population of *I. serracarajensis* exhibited a genetic diversity of 63.48% and a moderated level of intrapopulation genetic diversity (He = 0.187; Sh = 0.289; AMOVA, *P* = 55%). Despite moderate genetic diversity, sexual reproduction was not observed. Asexual reproduction by division of the corm through the emergence of individuals from the nonfertile mother plant is shown (Fig. S1). This phenomenon has been considered for *I. sinensis* and *I. chubutiana*, but no evidence was found (Hickey, Macluf & Taylor, 2003; Chen et al., 2009). Scanning electron microscopy studies of *I. serracarajensis* spores suggest that this species is probably polyploid (Pereira, Mittelbach & Labiak, 2015; Pereira et al., 2016), which may also explain the great diversity found in *I. serracarajensis*.

The maintenance of moderated diversity in *Isoetes serracarajensis* even after a process of population isolation is corroborated by the findings for other plant species. For instance, the *Orchidaceae Cattleya lobata* recently went through a process of population isolation and a decrease in size in Rio de Janeiro, Brazil. ISSR markers detected high intragenetic diversity ( *H* = 0.262; Sh = 0.463) of all the remaining populations, even populations under ecological stress, probably due to the maintenance of the ancestral genetic diversity of a historic moment of interpopulation connectivity and high gene flow (Gomes et al., 2018). In agreement, endangered angiosperm *Fritillaria imperialis* presented moderate levels of intrapopulation genetic variation (Sh: 0.61; 0.43; 0.36 for AFLP, ISSR and RAPD, respectively) and among populations (Gst = 0.63; 0,47; 0.41) (Badfar-Chaleshtori et al., 2012).

A high degree of genetic differentiation among populations accounted for approximately 54% of the total genetic diversity (G_ST_ = 0.544, AMOVA, P=45%), so both within-population diversity and diversity among populations contributed significantly to the total diversity of the species. Other species also showed high diversity among populations, such as *Argania spinosa* L. Skeels (Mouhaddab et al., 2017), *Brasenia schreberi* (Li et al., 2018), and *Pelatantheria scolopendrifolia* (Yun et al., 2020). Yun et al. (2020) showed that geographic isolation, low gene flow and asexual reproduction were related to the high divergence among *Pelatantheria scolopendrifolia* populations.

*I. serracarajensis* is found in several ferruginous plateaus with seasonally flooded environments. The rainy and dry seasons are well defined (Da Silva et al., 2018), and in the dry season (June to October), the species seems to disappear (few leaves resist air for an extended period) (Fig. S2), the corm is protected under the soil, and plants rise upon the beginning of the rainy season (November to May) (Fig. S2). These plants have mature spores before the reduction of soil humidity, and it is possible that this is the moment in which spores were dispersed, as reported by Taylor & Hickey (1992). For an amphibious plant, spore dispersion is more complicated and limited by the rainy season and other factors, such as wind (Troia, 2016; Ma et al., 2019).

The low gene flow (Nm = 0.419) found indicates that spore dispersion is reduced among populations (Table 3). Populations of *I. serracarajensis, IN3*, *N6*, *ISV*, and *IS11*, were grouped by Nei’s genetic distances, and a correlation was observed between genetic and geographical distance according to the Mantel test (Mantel, 1967). The analysis of genetic diversity in a spatial context can provide new insights into the understanding of the mechanisms of maintenance and dynamics of populations. The Mantel test has excellent applicability in conservation biology because it can help to effectively integrate genetic, demographic, and ecological processes (Escudero, Iriondo & Torres, 2003). The low estimated value of Nm (0.419) among *I. serracarajensis* populations and the significant correlation between geographical and genetic distances (Mantel *r* = 0.821, *P* < 0.0001) indicate that the geographical isolation barrier could principally affect gene flow. In fact, the Mantel test is reliable for determining the correlation between genetic and geographical distances among populations such as *Ottelia acuminate* var *jingxiensis* (Li et al., 2019) and *Brasenia schreberi* (Li et al., 2018). Spores of *Isoetes* are scattered gradually through the decay of sporangial tissue, and the proximity of megasporangia and microsporangia enables significant intergametophytic selfing analogous to self-pollination in seed plants (Caplen & Werth, 2000), which could contribute to lower genetic diversity in neighbouring plants and greater genetic diversity in distant plants, as shown for the endangered *Isoetes hypsophila* Chen et al. (2005).

## Conclusions

This work aimed to evaluate the genetic diversity of two species: one critically endangered (*I. cangae*), and the other classified as vulnerable (*I. serracarajensis*). Both species have high genetic diversity. The knowledge gained from the current study represents an indispensable prerequisite for effective conservation programmes (in situ and ex situ strategies) for both species since maintaining genetic diversity reduces extinction risk. Intra- and inter-region translocations are appropriate for in situ conservation strategies when conflict exists between conservation and economic interests. Translocations to the same or between management units are recommended to alleviate the impact of inbreeding and genetic drift. Interregional translocations must be performed very carefully because environmental differences can result in adaptive diversity loss. Additionally, ex situ conservation is also an important species maintenance procedure. The genetic diversity stock can be kept in active germplasm banks and spore banks. The source population(s) can be used to produce new plants to reintroduce into the natural environment if necessary. Our results provide valuable biological information for the conservation of the species and for future functional studies to uncover the physiological adaptations behind the endemic quillwort (*Isoetes* L.) species from Amazonian ferruginous outcrops to their specific environments. Detailed studies of ecological dynamics such as spore dispersion should be carried out to yield valuable information for further conservation of *I. cangae* and *I. serracarajensis*. Future studies on the effect of storage methods on spore viability will also be necessary.

##  Supplemental Information

10.7717/peerj.10274/supp-1 Supplemental Information 1 Photographs of sporophytes generation in *Isoetes* *Isoetes cangae* and *Isoetes serracarajensis* specimens. (A) *I. cangae* and *I. serracarajensis* (from left to right). (B) Young sporophytes generation from *in vitro* fecundation of *I. cangae*. (C) Asexual sporophytes generation (white arrow) from young plant nonfertile of *I. serracarajensis*. (D) Young sporophyte (white arrow) near of fertile adult plant of *I. cangae* observed in greenhouse.

10.7717/peerj.10274/supp-2 Supplemental Information 2 *I. serracarajensis* location on the ferruginous plateaus during rainy and dry seasons Figure S2. (A)* I. serracarajensis* location on the ferruginous plateaus of the southern portion marsh ISV during rainy and (B) dry seasons. (C) Detail of *I. serracarajensis* leaves during dry season (white arrows).

10.7717/peerj.10274/supp-3 Supplemental Information 3 Raw data from ISSR applied for data analyses and preparation for Tables 1–4 Gel results of ISSR primers, Mean Population Genetic Distance Matrix for Binary Distance, Results of Analysis of Molecular Variance.

10.7717/peerj.10274/supp-4 Supplemental Information 4 Raw data used for preparation of Figs. 2 and 3 Nei’s genetic and geographic distance matrices. Mantel test matrix.

## Additional Information and Declarations

### Field Study Permissions

The following information was supplied relating to field study approvals (i.e., approving body and any reference numbers): Collecting permits were granted by Instituto Chico Mendes de Biodiversidade do Ministério do Meio Ambiente (number 59724-2).

### Data Availability

The following information was supplied regarding data availability: The raw measurements are available in the Supplementary Files.
