# Supplementary figures and images for: The genetic diversity and population structure of two endemic Amazonian quillwort (Isoetes L.) species
Source: PeerJ. 2020 Nov 9;8:e10274. doi: 10.7717/peerj.10274 (PMC7659625; doi:10.7717/peerj.10274)

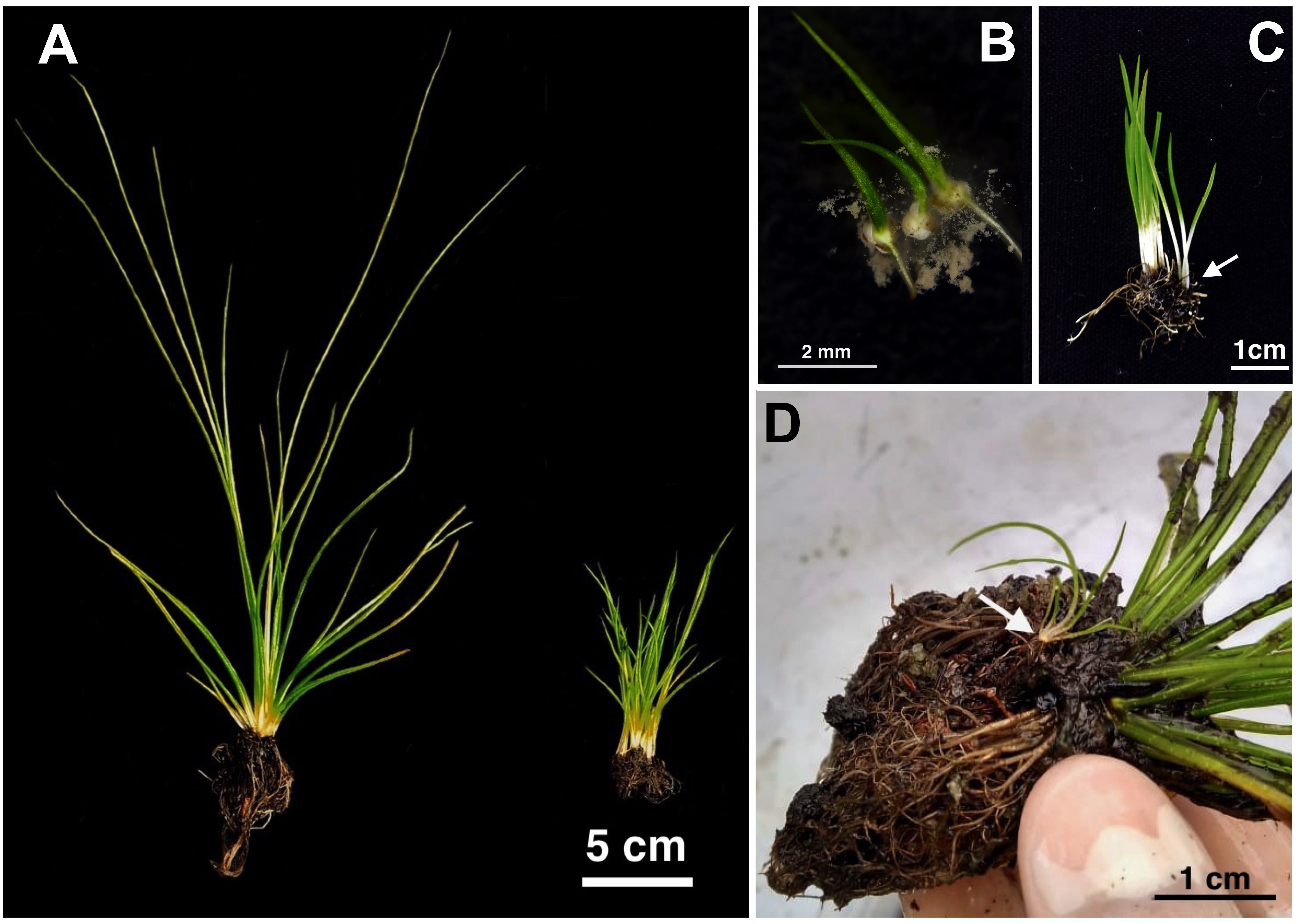

Supplement: Supplemental Information 1 — Isoetes cangae and Isoetes serracarajensis specimens. (A) I. cangae and I. serracarajensis (from left to right). (B) Young sporophytes generation from in vitro fecundation of I. cangae. (C) Asexual sporophytes generation (white arrow) from young plant nonfertile of I. serracarajensis. (D) Young sporophyte (white arrow) near of fertile adult plant of I. cangae observed in greenhouse. [file peerj-08-10274-s001.jpg]

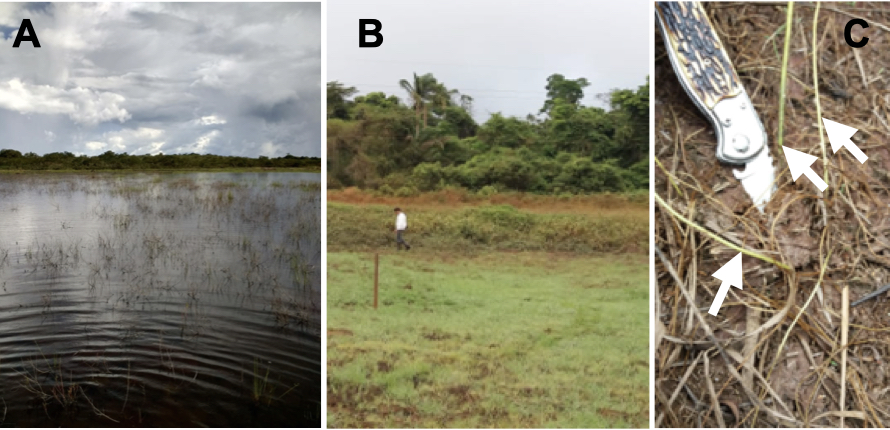

Supplement: Supplemental Information 2 — Figure S2. (A) I. serracarajensis location on the ferruginous plateaus of the southern portion marsh ISV during rainy and (B) dry seasons. (C) Detail of I. serracarajensis leaves during dry season (white arrows). [file peerj-08-10274-s002.jpeg]
